# Hybrid Antigens Expressing Surface Loops of ZnuD From *Acinetobacter baumannii* Is Capable of Inducing Protection Against Infection

**DOI:** 10.3389/fimmu.2020.00158

**Published:** 2020-02-07

**Authors:** Maryam Mobarak Qamsari, Iraj Rasooli, Somshukla Chaudhuri, Shakiba Darvish Alipour Astaneh, Anthony B. Schryvers

**Affiliations:** ^1^Department of Biology, Shahed University, Tehran, Iran; ^2^Molecular Microbiology Research Center, Shahed University, Tehran, Iran; ^3^Department of Microbiology, Immunology and Infectious Diseases, University of Calgary, Calgary, AB, Canada; ^4^Department of Biotechnology, Semnan University, Semnan, Iran

**Keywords:** protein engineering, ZnuD, hybrid antigen, *Acinetobacter baumannii*, protective epitope, outer membrane protein, lipoprotein, scaffold

## Abstract

*Acinetobacter baumannii* is an important human pathogen causing substantial mortality in hospitalized patients for which treatment with antibiotics has become problematic due to growing antibiotic resistance. In an attempt to develop alternative strategies for dealing with these serious infections surface antigens are being considered as targets for vaccines or immunotherapy. The surface receptor proteins required for zinc acquisition in Gram-negative bacterial pathogens have been proposed as vaccine targets due to their crucial role for growth in the human host. In this study we selected the putative ZnuD outer membrane receptor from *A. baumannii* as a target for vaccine development. Due to challenges in production of an integral outer membrane protein for vaccine production, we adopted a recently described hybrid antigen approach in which surface epitopes from the *Neisseria meningitidis* TbpA receptor protein were displayed on a derivative of the C-lobe of the surface lipoprotein TbpB, named the loopless C-lobe (LCL). A structural model for ZnuD was generated and four surface loops were selected for hybrid antigen production by computational approaches. Hybrid antigens were designed displaying the four selected loops (2, 5, 7, and 11) individually or together in a single hybrid antigen. The hybrid antigens along with ZnuD and the LCL scaffold were produced in the *E. coli* cytoplasm either as soluble antigens or as inclusion bodies, that were used to generate soluble antigens upon refolding. Mice were immunized with the hybrid antigens, ZnuD or LCL and then used in an *A. baumannii* sepsis model to evaluate their ability to protect against infection. As expected, the LCL scaffold did not induce a protective immune response, enabling us to attribute observed protection to the displayed loops. Immunization with the refolded ZnuD protein protected 63% of the mice while immunization with hybrid antigens displaying individual loops achieved between 25 and 50% protection. Notably, the mice immunized with the hybrid antigen displaying the four loops were completely protected from infection.

## Introduction

*Acinetobacter baumannii* is an opportunistic coccobacillus pathogen (1), classified as one member of the ESKAPE (*Enterococcus faecium, Staphylococcus aureus, Klebsiella pneumonia, A. baumannii, Pseudomonas aeruginosa, Enterobacter* spp.) group of pathogens by the Infectious Diseases Society of America (IDSA) (2). *Acinetobacter* infections such as septicemia, ventilator-associated pneumonia, bacteremia, urinary tract infections, wound sepsis, endocarditis, and meningitis have been observed in hospitalized patients. This nosocomial pathogen is considered as a common cause of hospital and community-acquired infections (3). Owing to the emerging multi and pan-drug resistance in this pathogen followed by subsequent problems in treatment (4, 5), WHO classified *A. baumannii* as one of the main three priority antimicrobial resistant pathogens that warrant investigation of new therapeutic approaches (5).

Vaccines are an alternative measure for fighting this ubiquitous opportunistic pathogen and reducing the morbidity and mortality in a particular population of patients. Several vaccine candidates including whole cells, outer membrane vesicles (6), and capsule components (7) have been tested. However, issues regarding safety, acceptability and ability to provide comprehensive protection against different strains of *A. baumannii* may limit the development of these as vaccine products (8). Recognizing the potential of surface protein antigens as potential vaccine antigens, *in silico* methods that are largely based on the first step of the reverse vaccinology approach (9, 10) have been used to identify potential vaccine candidates (8, 11). However, the *in silico* approaches for reverse vaccinology, such as using the presence of a signal peptide to select for potential surface proteins, cannot predict surface accessibility. Thus, without strong homology with a known surface protein, it is difficult to predict the surface accessibility and the resulting potential of selected antigens to induce a protective immune response.

An alternate approach to reverse vaccinology is to select surface antigens that perform an essential function in the host, such as the acquisition of transition metals ions (12). Transition metal ions, such as iron and zinc, serve as gene regulation, cellular metabolism and virulence factors (13) and cofactor for proteins that perform essential cellular processes (14). The approach of selecting essential surface proteins is particularly attractive if they interact with host proteins (15), as this ensures surface accessibility to immune effector mechanisms, and has been shown to provide effective protection from infection of the native vertebrate host (16).

In the vertebrate host, calprotectin (S100A7), a member of the S100 family of proteins, that binds zinc and manganese, has been implicated in the defense against infection by chelation of zinc (12). Eric Skaar's group performed a comprehensive study on the role of zinc acquisition by *A. baumannii* that focused on the impact of calprotectin on growth and the ability of *A. baumannii* to cause infection (14, 17). Inhibition of *in vitro* growth of *A. baumannii*, reversed by the addition of zinc, was used as an assay to detect transposon mutants defective in zinc acquisition. The authors identified a mutant in the cytoplasmic membrane permease (ZnuB) of a periplasm to cytoplasm transport system for zinc (ZnuABC). They subsequently screened for sequences with homology to the binding site for the Zur repressor protein, and demonstrated binding of Zur by a gel shift assay, identifying three additional putative transport genes regulated by zinc availability (*znuD1, znuD2*, and *TonB1*). The two orthologs of the *N. meningitidis* ZnuD protein (18, 19) shown to be involved in zinc transport, ZnuD1, and ZnuD2, were encoded by genes on the chromosome (A1S_2892) and a plasmid (A1S_3475) of *A. baumannii* strain ATCC17978, respectively. Recently, Eric Skaar's group characterized ZnuD1, now designated as ZnuD, in *A. baumannii* by demonstrating that a mutant strain with a *znuD* knockout was defective in growing in zinc restricted media, which was restored with either the addition of zinc chloride or complementation by expression of ZnuD in *trans* (20). Additional work to clarify the role of ZnuD2 in zinc acquisition in *A. baumannii* is required. Presumably these two proteins. ZnuD and ZnuD2 are orthologs of the TonB-dependent receptor protein TdtJ in *N. gonorrhoeae* and not the orthologs of the TdtH protein, that was originally thought to contribute to heme acquisition, but has now been shown to bind calprotectin and overcome calprotectin-mediated growth inhibition (13).

Although the TonB-dependent receptor proteins involved in nutrient acquisition may be ideal targets for vaccine development, there are considerable challenges in the commercial production of integral outer membrane proteins as they are normally produced in a membrane environment and require detergents or other lipophilic agents for solubilization and maintenance of structure. In addition, the important surface epitopes that would be the target of effective antibodies are formed by the extracellular loop regions of the beta-barrel, thus it is desirable to focus the immune response on those regions. These considerations provided the impetus for developing a hybrid antigen approach in which four surface epitopes of the integral outer membrane protein TbpA (transferrin binding protein A) from the human pathogen *Neisseria meningitidis* were displayed on the handle and barrel regions of the C-lobe derived from the surface lipoprotein TbpB (transferrin binding protein B) (21). The first step involved the removal and replacement of four extracellular loop regions of the C-lobe not resolved in the crystal structure of the *N. meningitidis* TbpB to create a loopless C-lobe (LCL) that served as a scaffold for displaying epitopes. A hybrid LCL-loop 10 antigen displaying the extracellular loop 10 of TbpA was capable of inducing antisera that was equivalent to or better than antisera directed against native, functional TbpA in binding to intact cells, blocking growth dependent upon exogenous transferrin and mediating complement mediated killing (21).

We decided to use this foreign scaffold for display of epitopes from *A. baumannii* ZnuD as the scaffold would not be expected to induce any antibodies against *A. baumannii* that could complicate the analysis of results. We used a series of bioinformatics approaches to identify potential epitopes that were incorporated into a foreign scaffold derived from the C-lobe of transferrin binding protein B (TbpB) from *N. meningitidis* to generate stable hybrid antigens (21). The hybrid antigens were used in immunization and challenge experiments to determine the nature and effectiveness of the immune response against the predicted epitopes and then mapped onto a computational model of the ZnuD structure.

## Materials and Methods

### Computational Modeling, Loop Selection, and Design of Hybrid Antigens

**Figure 1:**
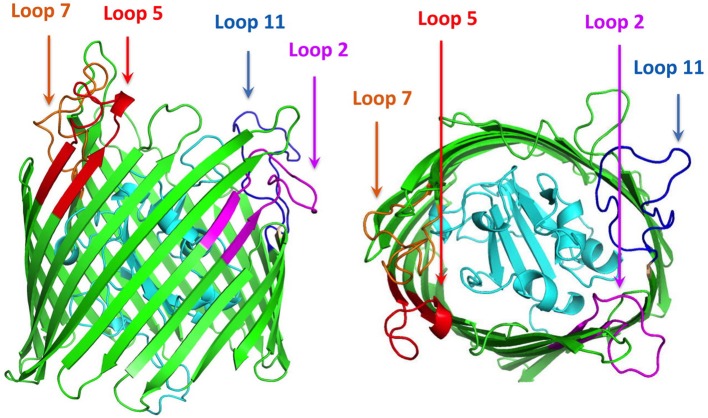
Cartoon diagram of a computational structural model of ZnuD from *A. baumannii*. The plug region is colored in cyan and the beta-barrel is colored green. The regions of the four loops selected for display on the LCL scaffold are labeled and colored in representing the four predicted B-cell epitopes. Loop 2–purple, Loop 5–red, Loop 7–orange, and Loop 11–blue.

Using the ZnuD sequence available on the NCBI web site (accession number eex02556.1) a variety of bioinformatics analyses were used to probe the predicted secondary structural features of the protein. After removing the predicted signal peptide region with the SignalP program (22), computational modeling approaches were used to develop three dimensional models for ZnuD including I-Tasser (23) and RaptorX (24). The position of the beta strands in the 22-strand beta-barrel was virtually identical in I-Tasser and Raptor-X models, indicating that the “anchoring position” of the extracellular loops of this TonB-dependent outer membrane antigen were identical (Figure 1).

A search for potential B-cell epitopes in ZnuD was performed using nine different servers, the ABCpred server (25), the SVMTriP server (26), the CBTOP server (27), the Ellipro server (28), the LBTope server (29), the BepiPred-2.0 server (30), DiscoTope 2.0 server (31, 32), the IEDB server (33), and the BC pred server (25). The results were aligned and the loops that were identified by at least 5 servers were selected for further analysis (Table 1). Loops were narrowed down based on criteria identifying epitopes encompassing a majority, if not all of, the loop region and were at least 20 amino acids long, as prior experiments demonstrated the bigger loops were functionally more effective (21). With these paraments, loops 2, 5, 7, and 11 were identified and selected for “loop transplant” (Figure 1).

**Table 1:** Analysis of B cell epitope server results and loop selection for loop transplant.

**Loops**	**Number of servers identifying epitopes**	**Number of servers identifying 85% of loop as an epitope**	**Loops over 20 amino acids**
Loop 1	3/9	3/9	No
Loop 2	8/9	5/9	Yes
Loop 3	6/9	2/9	Yes
Loop 4	4/9	1/9	Yes
Loop 5	6/9	6/9	Yes
Loop 6	5/9	0/9	No
Loop 7	8/9	5/9	Yes
Loop 8	2/9	1/9	Yes
Loop 9	7/9	2/9	Yes
Loop 10	5/9	5/9	No
Loop 11	8/9	4/9	Yes

*The highlighted bars represented loops that have the necessary requirements for transplant*.

**Figure 2:**
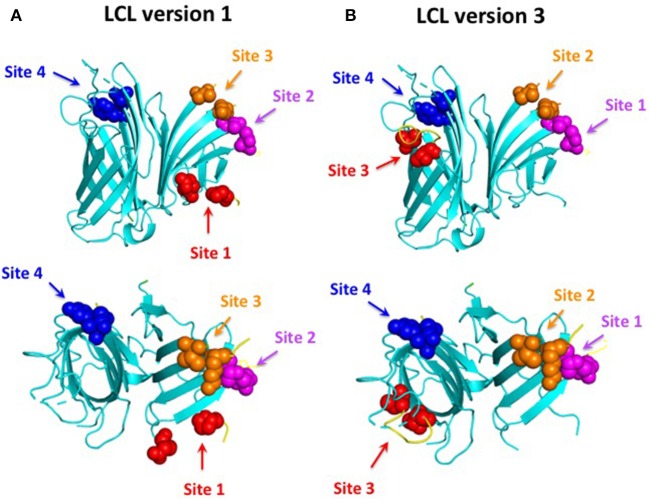
Comparison of LCL V1 **(A)** and LCL V3 **(B)**. Cartoon diagram of the LCL scaffold derived from the crystal structure of the *N. meningitidis* C-lobe (PDB 3VE2) with the amino acids flanking the four insertion sites labeled as colored spheres. LCL V3 was used to display the four predicted B-cell epitopes. Site 1 (loop 21) on LCL V3 hosted loop 2 from ZnuD, site 2 (loop 23) hosted loop 5, site 3 (loop 27) hosted loop 7, and site 4 (loop 31) hosted loop 11.

The original “loopless C-lobe” (21) was derived from the *N. meningitidis* TbpB from strain M982 in which the larger loop regions not resolved in the crystal structure (loop 20^413−439^, loop 21^444−474^, loop 23^499−520^, loop 31^657−676^) (34) were replaced with equivalent short linking regions identified in the structure of an *Actinobacillus pleuropneumoniae* TbpB (35). Two variants of the original LCL were prepared which involved the replacement of a segment of the handle domain (LCLv2) or loop 31 (LCLv3) were derived from the TbpB from *N. meningitidis* strain B16B6 (36) to generate a more cross-protective scaffold when used in experiments with *N. meningitidis*. The M982 LCL with the loop region derived from the B16B6 TbpB (loop 31^657−676^), LCLv3, was used as the starting scaffold for hosting the loops from *A. baumannii* ZnuD and is localized to insertion site 4 (Figure 2). The LCL v3 used in the current study had foreign loops inserted into sites 1–4, created by replacing loops 21, 23, 27, and 31 from the scaffold. ZnuD extracellular loop 2^175−196^, loop 5^305−325^, loop 7^383−406^, and loop 11^552−576^ (Figure 1) were selected for insertion into sites 1, 2, 3, and 4 of LCLv3, respectively (Figure 2). Site 1 in the original LCL (21) is not flanked by typical anti-parallel beta strands as it was originally designed to accommodate the loop 3 helix region from TbpA, thus an additional site (loop 27^588−594^) flanked by beta-strands was selected for display of foreign loops in LCLv3 (Figure 2).

### Bacterial Strains and Plasmids

*Acinetobacter baumannii* ATCC 19606 was used for the sepsis experiments and as a source of DNA for the *znuD* gene. *Escherichia coli* strain Top10 was used for the cloning of the hybrid antigen genes and strain ER2566 was used for the production of recombinant antigens. The bacteria were grown in Luria Bertani (LB) broth or on LB agar medium at 37°C. For strains carrying plasmids growth media were supplemented with ampicillin (100 μg/ml). Bacterial stocks were maintained in 30% glycerol at−80°C for long term storage.

A custom expression plasmid (e5044) with the T7 promoter, an N-terminal polyhistidine tag, maltose binding protein and TEV protease cleavage site followed by *Bam*H1 and *Xba*I/*Hind*III cloning sites was used for production of the recombinant proteins.

### Production of ZnuD and the Hybrid Antigens

The coding sequence of *znuD* was amplified from the genomic DNA of *A. baumannii* ATCC 19606 and sequenced. The mature coding sequence of the *znuD* gene was amplified with the primers listed in Table 2, digested with BamHI and XbaI and cloned into the custom expression plasmid e5044. The gene encoding the Nm LCLv3 flanked by BamHI and XbaI sites were also cloned into this expression plasmid for production.

**Table 2:** Primers sequences used in this study.

**Amplicon name**	**Primer sequence**	**Amplicon size (bp)**
ZnuD	F: AGTGCA**GGATCC**GAAGATGCAACAAATGCAAC	1850
	R: TGCACA**TCTAGA**TTAGTAGCTTAAGGTCAC ACCAGC	
LCLv3	F: GTCGTCAGACTGTCGATGAAGCC	111
	R: ATGAGGCTGCGGTTGCGTTCCTG	
Loop 2^204−227^	F: ACCCGCAAATTTGAACACACGcgttttgaaacagatggt	
	R:ACAGCAGACTTCGACTTCATAGGTtttttgatcaaatccagc	
Loop 5^335−354^	F: TACGGAATGTTGACGCGCAAAaaaacaactgcagagaaagt	108
	R: TGGAGGAACATACTTTGTTCAACcttattagaattatcgaatcc	
Loop 7^412−438^	F: AAAATTACCGGTAAACTGACGcgtgcaccagttgtaggt	114
	R: CATGCCTTCAATCGTAAAGGTttctggttttaaatctgg	
Loop 11^583−606^	F:GGCGGTTGGTTTGCGTATcaaaacctatatgatacagactataagactgc	117
	R: GCACCGAAGACGACCGTTGCaaggcggccgctggcaat	
Position 1	POS1R: accatctgtttcaaaacgCGTGTGTTCAAATTTGCGGGT	290
	POS1F: gctggatttgatcaaaaaACCTATGAAGTCGAAGTCTG	708
Position 2	POS2R: actttctctgcagttgttttTTTGCGCGTCAACATTCCGTA	370
	POS2F: ttcgataattctaataagGTTGAACAAAGTATGTTCCT	633
Position 3	POS3R: acctacaactggtgcacgCGTCAGTTTACCGGTAATTTTCTTG	580
	POS3F: ccagatttaaaaccagaaACCTTTACGATTGAAGGC	408
Position 4	POS4R: tgtatcatataggttttgATACGCAAACCAACCGCC	790
	POS4F: attgccagcggccgccttGCAACGGTCGTCTTCGGT	190

*Small letters on primer sequences correspond to loop sequences and capital letters correspond to LCL positions. Bold and underlined text indicates a BamH1 and Xba1 site in the forward and reverse primers respectively*.

The DNA regions encoding the *znuD* loops 2, 5, 7, and 11 were inserted into LCLv3 at the four selected sites (Figure 2) by SOE (splicing by overlap extension)-PCR (37) using the primers listed in Table 2. The hybrid antigen gene with all four loops was created by successive SOE-PCR reactions to introduce the individual loops. Colony PCR with loop and vector specific primers were used to identify positive colonies, and subsequently the genes were amplified and confirmed by sequencing.

ZnuD, Nm LCLv3, and the ZnuD- Nm LCLv3 hybrid proteins were expressed by growth in autoinduction medium (38) and the bacteria from overnight cultures were pelleted by centrifugation (10,000× g/20 min/4°C). The pellets were subsequently suspended in TE lysis buffer (Tris-HCL 10 mM, EDTA 1 mM) and sonicated on ice for 20 min (with 70% resonance power at 30 s interval). The suspension was pelleted by centrifugation (10,000× g for 20 min at 4°C) and Ni-NTA agarose was used to capture soluble recombinant proteins. The proteins were eluted by an imidazole containing buffer, dialyzed against phosphate buffered saline and analyzed with a 12% acrylamide SDS-PAGE gel, stained by Commassie Brilliant Blue dye.

For those proteins that were not readily detectable in the SDS-PAGE gel, a procedure for isolation of protein from inclusion bodies was implemented. The pellet was re-suspended in buffer B (NaH2PO4 100 mM, NaH2PO4 10 mM, Urea 8 M, pH = 8.0) and incubated on ice for 15 min. After centrifugation (10,000× g for 20 min at 4°C) to remove debris, the solubilized inclusion body protein was captured with a Ni-NTA agarose column. The column was then exposed to buffer with decreasing concentrations of urea from 6 to 2 molar. The protein was then eluted from the column with buffer containing 2 M urea and 300 mM imidazole and then dialyzed against PBS buffer. The dialyzed samples were subsequently analyzed with a 12% SDS-PAGE gel, stained by Commassie Brilliant Blue dye.

### Animal Study and Ethical Clearance

Six to eight-week-old healthy male BALB/c mice weighing 20–25 g were procured from the Razi Institute, Tehran. Iran. Mice were housed in hygienic standard and well-aerated conditions in the animal care facility at Shahed University. All the animal tests were performed in accordance with animal care guidelines confirmed by Animal Care and Ethical Committee of Shahed University.

### Mouse Immunization and Antibody Titer Measurement by ELISA

Fifteen mice were selected for immunization with each protein. The mice were immunized subcutaneously (SC) with 20 μg protein three times at 2-week intervals on days 0, 14, and 28. 100 μl of the protein-adjuvant mixture at 1:1 (v/v) ratio was administered to mice. Freund's Complete adjuvant (Sigma) was used at the first step followed by the use of the incomplete adjuvant at the second and third booster shots. The sera samples were collected 1 week after each dose.

IgG production against each protein was measured by enzyme-linked immunosorbent assay (ELISA). For the ELISA assay 96 well flat-bottom plates were coated with 5 μg/well of each antigen in carbonate-bicarbonate buffer (pH 9.6) at 4°C overnight. The plates were washed three times with PBS containing 0.05% Tween 20 (PBST) and blocked using 5% skim milk (w/v) in PBST at 37°C for 45 min. After washing, serial dilutions of sera from 1:250 to 1:128,000 were added in duplicates and incubated at 37°C for 2 h. The plates were washed and then 100 μl of 1:15,000 dilution of horseradish peroxidase-conjugated goat anti-mouse IgG was added to each well. Following 1 h incubation of the plates at 37°C and washing, 3,3′,5,5′-tetramethylbenzidine (TMB) solution was added and incubated in dark for 20 min at room temperature. 3M H2SO4 was used to stop the reaction followed by absorbance measurement at 450 nm on ELISA plate reader.

### *A. baumannii* Sepsis Model

The lethal dose (LD100) of *A. baumannii* ATCC 19606 was determined by infecting groups of 8 mice with ascending bacterial concentrations from 10^4^ to 10^7^ CFU in 100 μl phosphate-buffered saline, supplemented with porcine mucin. The preparation of bacterial inoculum for administration was according to Harris et al. (39). Briefly, *A. baumannii* ATCC 19606 was grown for 18–24 h in 100 ml LB broth medium and adjusted to the appropriate concentration in 250 μl physiological saline. The bacterial-saline solution was mixed with 10% (V/V) porcine mucin (Sigma-Aldrich) in saline at a ratio of 1:1. The final inoculum was a 500 μl bacterial-saline solution with 5% (V/V) porcine mucin.

For the active immunizations experiment, groups of 8 mice immunized were challenged via the intraperitoneal route 3 weeks after the last immunization with a lethal dose of *A. baumannii* ATCC 19606, which was determined to be 1.8 × 10^6^ CFU. The mice were monitored for 168 h post challenge for clinical symptoms and euthanized when severely sick or at experimental endpoint.

Sera collected from the immunized groups of 8 mice just prior to challenge in the active immunization study was pooled for use in the passive immunization study. The sera was heat treated at 65°C for 30 min to inactivate endogenous complement activity. Groups of 6 unimmunized mice aged 49 days were injected intravenously with 100 μl of the heat inactivated sera 3 h prior to an intraperitoneal challenge with the lethal dose of *A. baumannii* ATCC 19606. As with the active immunization study, the mice were monitored for 168 h post challenge and euthanized when severely sick or at experimental endpoint.

### Evaluation of Bacterial Burden in Mice Organs and Cytokine Analysis

Groups of 3 mice were immunized with the different antigen formulations, challenged with *A. baumannii* ATCC 19606 and the bacterial load determined in the lungs, livers, and spleens. Mice were immunized and challenged intraperitoneally with 1.0 × 10^6^ CFU of *A. baumannii* ATCC 19606 as previously described above. The animals were sacrificed 24 h after challenge and the lung, liver, and spleen were removed aseptically. Tissues were weighed and then homogenized in 2 ml of physiological saline. Serial dilutions of the homogenates were plated onto LB agar and incubated at 37°C for 18–24 h. The number of colony forming units were determined and the results were expressed as log10 CFU.

For cytokine analysis, serum samples were taken 24 h after the bacterial challenge and prior to the tissue collection for the bacterial load analysis to determine levels of TNF-α, IFN-γ, and IL-10 in the blood. Cytokine levels were estimated using a sandwich ELISA method using kits from Krishgen Biosystems, India and GenAsia, Philippines according to the supplier's instructions.

### Statistical Analyses

Differences in quantitative measurements were assessed by one-way and non-parametric analysis of variance (ANOVA) followed by either Duncan's Multiple Range Test or Tukey's *post hoc* Analysis with PRISM 8.0 Graph pad software. Non-parametric Log Rank test was used to compare the survival rate. Differences were considered significant at *p* < 0.05. The data were presented as mean with standard deviations.

## Results

### Structural Modeling and Identification of Epitopes

The first step in selection of epitopes was to develop a structural model for the *A. baumannii* ZnuD using computational approaches so that the surface exposed loops of this TonB-dependent outer membrane protein could be identified. Although a variety of computational approaches were used for comparison, the output from the I-Tasser was ultimately used for the structural model (Figure 1). As a means of potentially selecting the best loops for display on the hybrid antigen scaffold, a series of B-cell epitope prediction programs were used (see Materials and Methods section) and the results compared to identify the top four scoring peptide regions. These peptide regions overlapped extracellular loop 2, 5, 7, and 11 (numbered from the N-terminus) and were used to design the loop regions to be transplanted on to the hybrid antigen scaffold.

### Preparation of Hybrid Antigens

In order to be able to present the four selected surface loops to the immune system we opted to display them as surface loop regions on a foreign scaffold derived from the TbpB C-lobe from *N. meningitidis* (21). The scaffold is comprised of anti-parallel beta-strands that form an N-terminal handle domain and a C-terminal barrel domain with accessible connecting “loop” regions suitable for display of the external loop regions from integral outer membrane proteins (Figure 2). The original derivative of the *N. meningitidis* TbpB C-lobe was termed the loopless C-lobe (LCL) (21) as it had removed the loop regions not resolved in the crystal structure of TbpB (34). Since the original scaffold used one site for displaying the loop 3 alpha-helix region that is not optimal for displaying loop regions flanked by anti-parallel beta-strands, when we used a modified version of the LCL in this study an alternate site was used for loop display (Figure 2). It is important to note that the reason we used a “foreign” scaffold is that it is unlikely to induce antibodies against *A. baumannii* thus any sera reactivity or ability to protect against infection can be attributed to the displayed epitopes.

Primers (Table 2) were designed to the extracellular loop regions from ZnuD with the 5′ regions corresponding to the four selected sites on scaffold. The loops were introduced into the four sites of the LCL by SOE PCR (37) so that they would encode hybrid antigens containing the individual loop regions (LCL-loop 2, LCL-loop 5, LCL-loop 7, LCL-loop 11) or one containing all four epitopes (LCL-loop 2, 5, 7, 11). The genes encoding the LCL control and the hybrid antigens were cloned into a custom expression vector encoding an N-terminal polyhistidine tag fused to the maltose binding protein (Mbp) followed by a TEV protease cleavage site with a T7 promoter driving the expression of the recombinant hybrid protein. The DNA region encoding the mature ZnuD protein was also cloned into the custom expression vector for cytoplasmic production.

The T7 promoter mediated expression system is designed for very high-level protein expression that is only attained with a limited number of genes, as the expression of many genes result in a balance between soluble protein and inclusion bodies due to overexpression. Thus, when the vectors containing the genes encoding the LCL, hybrid proteins and ZnuD were transformed into a host strain with an inducible T7 RNA polymerase and grown overnight on auto-induction medium (38) only the vectors containing the genes encoding LCL or hybrid proteins displaying loops 2 and 7 provided sufficient soluble protein in the supernatant of lysed cells for subsequent experiments. For the remaining genes encoding hybrid antigens or the ZnuD protein, the inclusion bodies in the cell pellet were solubilized in buffers containing 8 M urea and the recombinant proteins purified with a Ni-NTA Sepharose column, analogous to the soluble hybrid proteins. The proteins solubilized from inclusion bodies were refolded by using a gradient of 6–2 M urea. The urea concentration was further reduced by dialysis against PBS.

**Figure 3:**
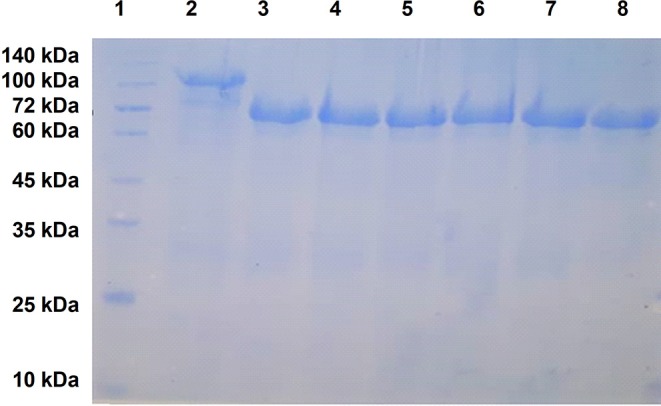
SDS-PAGE analysis of purified recombinant ZnuD and the hybrid antigens. Note that all antigens contain a N-terminal HisMbpTEV fusion partner thus are 40 kDa larger than the isolated protein antigen. Lanes; 1–MWS, 2–ZnuD, 3–Loop 2, 4–Loop 5, 5–Loop 7, 6–Loop 11, 7–Loop 25711 (all loops), 8–LCL.

As illustrated in Figure 3, the recombinant fusion proteins with ZnuD or the various hybrid proteins were relatively pure, migrating at ~105 kDa and between 70 and 75 kDa, respectively, due to the 40 kDa Mbp fusion partner. Although the option of performing proteolysis with a TEV protease preparation to remove the Mbp fusion partner was considered, we opted to perform the immunization and challenge experiments with the purified fusion proteins to avoid the not infrequently observed loss of protein. Since our primary objective was to observe the immune response due to the displayed epitopes, the fusion protein containing LCL serves as a negative control to account for any effects of the fusion protein.

### The Immunogenicity of the Hybrid Antigens

**Figure 4:**
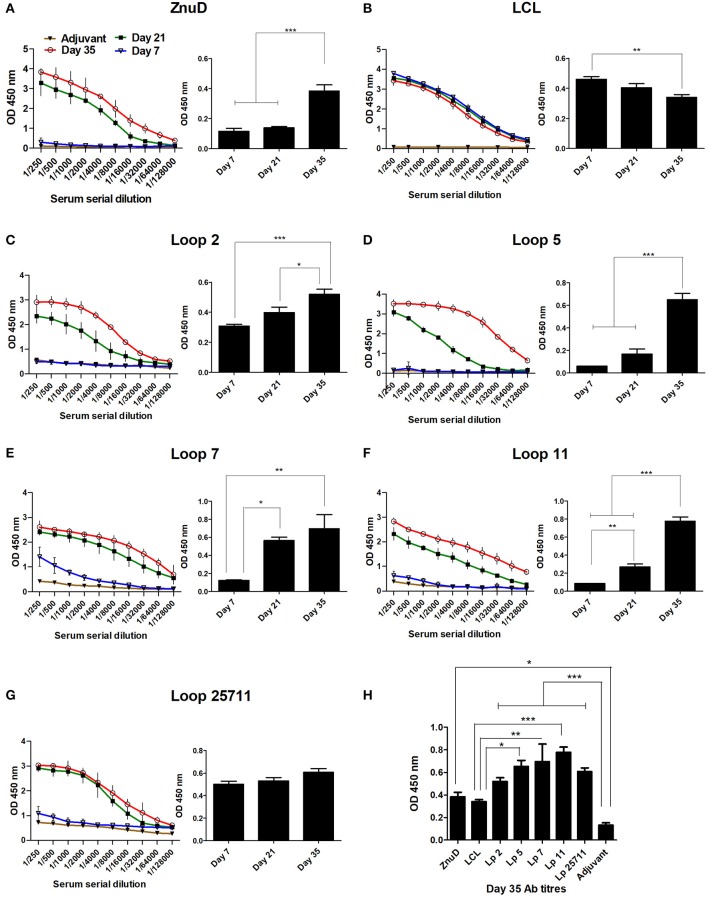
Serum collected from the immunized mice were analyzed for IgG titres against the immunizing protein. **(A–G)** Display the absorbance values (OD 450) at two-fold serial dilutions of the serum and the absorbance value at the highest dilution tested (1/128,000) at days 7, 21, and 35. **(H)** The absorbance value for all sera tested at the dilution 1/128,000 at day 35. Sera from mice from each immunization group (*n* = 6) were assayed in duplicate at each time-point. The adjuvant control group (*n* = 6) were assayed at the last time-point. Significance is displayed as ^*^p < 0.05, ^**^p ≤ 0.01, and ^***^p < 0.001.

Groups of 15 mice were immunized three times at 2-week intervals and the sera was collected and analyzed 7 days after each immunization prior to using the mice in the challenge studies, for cytokine analyses or evaluation of bacterial load in the tissues. Immunogenicity of the different vaccine formulations were assessed by measuring total IgG titres in the serum at D7, D21, and D35 and are displayed as absorbance values (OD450) at 2-fold dilutions of the serum (Figure 4). The absorbance observed at the highest dilution (1:128,000) is also demonstrated. Notably, the control LCL antigen achieved high titres after the first immunization that was not substantially enhanced after the second and third immunization (Figure 4B), a phenomenon not observed with the hybrid antigens (Figures 4C–G). Since it was not observed with the hybrid antigen formulations it could not be due to the presence of Mbp or LCL epitopes that would be readily accessible on the hybrid antigens. Whether the greater stability of LCL relative to the hybrid antigens or interference with access to epitopes on the surface that displays the loops contributes to this phenomenon is uncertain. Nevertheless, this antibody response had no protective effect against *A. baumannii* infection (Figure 4) thus did not impact the major findings of this study.

In contrast, substantial titers were not achieved until the second immunization for ZnuD and all of the hybrid antigens displaying loops (Figures 4A,C–G). Although there was some variation in the background readings for the control adjuvant, possibly due to minor contaminants in the preparations used to coat the ELISA plates, all the antigens achieved reasonably high antibody titers (>1/32,000) by the third immunization. Figure 4H displays the absorbance values at Day 35 for the highest dilution factor tested (1:128,000). All antigens induced an immunogenic response compared to the control.

### The Protective Effect of the Immune Response Against the Selected Epitopes

**Figure 5:**
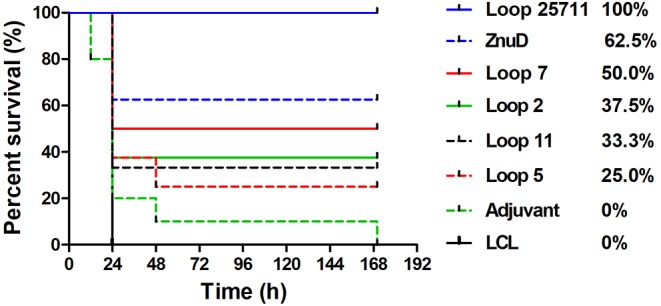
Survival curves of mice immunized with different antigens and challenged by *A. baumanii* ATCC 19606. Mice were challenged with 1.8 × 10^6^ bacteria and monitored for 168 h (7 days) post challenge for symptoms.

Eight mice from each of the groups of mice immunized with various antigens and adjuvant control were selected for challenge with an intraperitoneal injection of a lethal dose of *A. baumannii* strain ATCC 19606. Unfortunately, two mice in the group immunized with loop 11 were inadvertently removed prior to challenge, thus only 6 were available for the challenge experiment. Three weeks after the last immunization (D49) the mice were challenged with a lethal dose of *A. baumannii*. At this challenge dose the control mice treated with adjuvant and the mice immunized with the LCL did not survive (or were euthanized) by the end of the experiment (Figure 5). All of the mice immunized with the hybrid antigen displaying all four loops (2, 5, 7, and 11) survived until the end of the 7-day evaluation period. In the group immunized with recombinant ZnuD, 3 mice did not survive after the first day, but 5 of the mice survived until the end of the experiment. In the mice immunized with the hybrid antigens displaying individual loops mice that survived beyond day 1, survived until the end of the experiment with survival rates decreasing from the hybrid antigen displaying loop 7 (4/8), loop 2 (3/8), loop 11 (2/6), to loop 5 (2/8).

**Figure 6:**
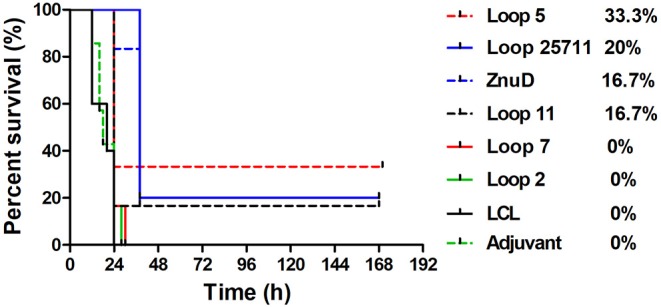
Survival curves of mice passively immunized with serum from mice immunized with different antigens and challenged by *A. baumanii* ATCC 19606. Mice were challenged with 1.8 × 10^6^ bacteria and monitored for 168 h (7 days) post challenge for symptoms.

In an attempt to determine whether the protective effect observed in the active immunization study was due to the presence of antibody, a passive immunization experiment was performed in which an intravenous injection of 100 μL of sera from mice immunized with the same set of antigens was made into groups of 6 naïve mice 3 h prior to intraperitoneal challenge with of a lethal dose of *A. baumannii* strain ATCC 19606 (Figure 6). A similar pattern where mice that survived after the first day or 36 h, survived until the end of the experiment was observed. Not surprisingly, the protection was not as effective as with the active immunization study with no protection observed for two of the hybrid antigens (loop 7 and 2), partial protection (1/6) with one of the hybrid antigens (loop 11) and the best protection observed with the hybrid antigen displaying loop 5 (2/6). In contrast to the active immunization study, sera from mice immunized with recombinant ZnuD did not provide superior protection than the single epitope hybrid antigens and the sera from mice immunized with hybrid antigen with four loops was not substantially superior to the others.

### The Impact on Bacterial Load in the Lungs, Spleen, and Liver

**Figure 7:**
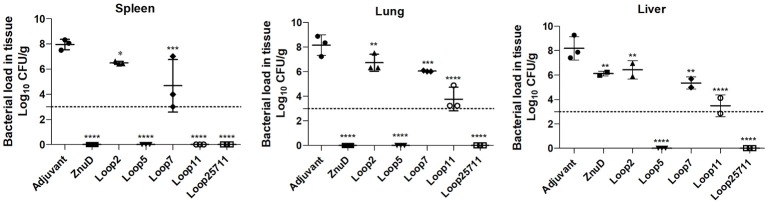
Evaluation of bacterial load in lung, liver, and spleen of immunized and control mice groups. Mice were challenged with 1.0 × 10^6^ bacteria and euthanized for tissue collection after 24 h. Significance is displayed as ^*^p < 0.05, ^**^p < 0.01, ^***^p < 0.001, and ^****^p < 0.0001 compared to the adjuvant group. The limit of detection is indicated by the dashed line.

The presence of bacteria in the lungs, spleen and liver 24 h after intraperitoneal challenge of a sub-lethal dose of *A. baumannii* was monitored by colony count (Figure 7) in 3 mice from each group. Since challenge studies with the lethal dose of *A. baumannii* ends up in rapid death of the most of infected animals, especially within control groups, a sub-lethal dose was used for both control and test groups to better monitor the animals post 24 h of infection.

The relatively high CFUs in the adjuvant control treated mice compared to the virtual absence of colonies in mice immunized with the hybrid antigen displaying four loops strongly suggests that the observed colony count from the lungs, spleen and liver are solely due to *A. baumannii*. The ability of the hybrid antigen displaying all four loops (2, 5, 7, and 11) to effectively remove *A. baumannii* from all the monitored tissues, is consistent with its ability to fully protect against lethal challenge after active immunization (Figure 5) and likely is through antibody mediated mechanisms. The relative lack of clearance of *A. baumannii* from the liver by ZnuD (Figure 7) and reduced protection against infection after active immunization relative to the four loop hybrid antigen (Figure 5), may be attributed to lack of fully-folded, functional protein, but underscores challenges in production of this antigen. The more effective removal of *A. baumannii* from the monitored tissues by the hybrid antigen displaying loop 5 than hybrid antigens displaying loop 11, and particularly loops 2 and 7 (Figure 7), does not correlate with the results from the active immunization study (Figure 5). Although it does provide some optimism regarding the potential utility of hybrid antigens displaying individual loops at inducing a protective immune response, caution should be exercised to not overinterpret the data, as any conclusions regarding the value of targeting specific loops would require more extensive experimentation.

### The Cytokine Response

**Figure 8:**
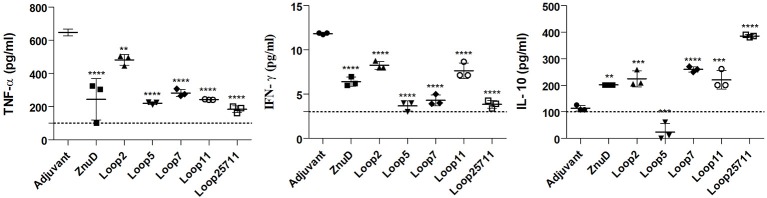
Serum collected from the immunized and control mice were analyzed for TNF-α, IFN-γ, and IL-10. Sera was collected 24 h after bacterial challenge with 1.0 × 10^6^ CFU. Significance is displayed as ^**^p ≤ 0.01, ^***^p < 0.001, and ^****^p < 0.0001 compared to the adjuvant group. The limit of detection is indicated by the dashed line.

In the mice used in the bacterial load study, serum samples were taken 24 h after challenge and before euthanizing the mice and sampling the tissues for bacterial load. The levels of three cytokines in the serum were measured (Figure 8). The pro-inflammatory (TNF-α) and inflammatory (IFN-γ) cytokine levels were reduced in all the mice immunized with protein antigens compared to the adjuvant control mice. Notably there were significant reductions in the level of TNF-α (*p* ≤ 0.01; *p* ≤ 0.0001) and IFN-γ (*p* ≤ 0.0001) for the mice immunized with the Loop 5 and all loop (2, 5, 7, 11) hybrid antigens, respectively. This correlates with the more effective clearance of bacteria from the tissues in these mice (Figure 8). In contrast, there was increased levels of the anti-inflammatory cytokine (IL-10) in all immunized infected mice except those immunized with the hybrid antigen displaying loop 5. The fact that the IL-10 levels in the mice immunized with loop 5 hybrid antigen and the all loop (2, 5, 7, 11) hybrid antigen were the two most divergent, underscores the fact that these levels did not correlate with clearance of the bacteria.

## Discussion

The failure to effectively deal with *A. baumannii* infections by conventional management and control approaches and the limited options for antibiotic treatment have prompted calls for considering novel research directions to explore other options such as vaccination strategies (40). In this era of readily available genomic sequences there are many options, tools and approaches that can be pursued for selection of potential vaccine targets for protein-based vaccines, but whether the proposed targets are expressed and accessible at the cell surface under *in vivo* conditions is a critical question that is not readily addressed. Since the availability of transition metals are limited within the host environment by a variety of host mechanisms (12), surface proteins involved in their acquisition are potential targets for vaccine development as their expression is required for survival in the host. The potential for this type of approach has been demonstrated by recent successes in providing protection from infection in the natural porcine host, with vaccine preparations targeting surface receptors involved in acquiring from the host iron-binding protein transferrin (16, 41). The rationale for targeting surface proteins involved in iron acquisition also underlies recent attempts to target the BauA siderophore receptor from *A. baumannii* (42, 43).

The identification of surface receptors required for zinc acquisition in *A. baumannii* (17) provides additional targets expected to be expressed *in vivo*, particularly at sites of inflammation where zinc is sequestered by the host protein calprotectin. Studies in *N. meningitidis* identified a surface receptor involved in zinc acquisition, ZnuD, that was suggested to be a candidate for a universal meningococcal vaccine (18, 44). Subsequent structural studies not only provided detailed structural information on this TonB-dependent transport protein, comprised of a 22-strand anti-parallel beta-barrel with a central plug domain, but demonstrated that the conformation of the external surface loops of the barrel were modified by the presence of zinc (19). In addition to the ZnuD protein in *N. gonorrhoeae*, that has been referred to as TdfJ, a second surface receptor involved in zinc acquisition has been identified, TdfH, that mediates binding of calprotectin by gonococcal cells (13). The two zinc receptors in *A. baumannii* ATCC17978, ZnuD (ZnuD1 initially) and ZnuD2, were identified by the presence of a consensus binding site for the zinc-dependent regulatory proteins Zur, and annotated in the genome as TonB-dependent receptors (17). Erik Skaar's group have further shown *in vitro* that ZnuD is necessary for the internalization of zinc in low zinc conditions (20). Since the gene for one of the proteins (ZnuD) is on the chromosome and the other (ZnuD2) is on a plasmid, it seemed logical to target the chromosomally encoded protein that is likely to be common to all *A. baumannii* strains.

TonB-dependent receptor proteins involved in nutrient acquisition are ideal targets for vaccine development however they are notviable as commercial vaccines on their own due to challenges in the production of integral outer membrane proteins. It is also desirable to focus the immune response on important surface epitopes formed by the extracellular loop regions of the beta-barrel, that would likely be the target of effective antibodies. These considerations led to the utilization of a hybrid antigen approach where we decided to use a foreign scaffold for display of epitopes from *A. baumannii* ZnuD.

Since there are no established approaches to determine which surface loops of TonB-dependent receptors are suitable for inducing an effective immune response against the targeted Gram-negative pathogen, we decided to use established bioinformatics approaches to select the best B-cell epitopes in ZnuD from *A. baumannii*. Ten different bioinformatics servers that predict potential B-cell epitopes were used to select the optimum epitopes (25–33, 45) and four epitopes with the highest scores were selected after aligning the results from the different servers. These peptide regions mapped to loops 2, 5, 7, and 11 of ZnuD (Figure 1) thus the DNA encoding these loop regions were inserted into gene encoding a modified version of the *N. meningitidis* loopless C-lobe (LCL) at four sites so that the resulting hybrid gene displayed the ZnuD loops on the four insertion sites on LCL (Figure 2).

Although four of the recombinant antigens (loop 5, loop 11, all loops and ZnuD) did not rapidly fold into functional protein but instead formed large aggregates of misfolded protein as inclusion bodies, they were able to be refolded into a soluble form after solubilization in 8 M urea. This is not surprising for the hybrid antigens since the high level of expression with the T7 promoter is not optimal for most proteins, and they would naturally for a soluble antigen upon refolding. However, since ZnuD is an integral outer membrane protein it would not naturally fold into is native conformation in an aqueous solution without the presence of detergents or other amphiphilic molecules, as the *N. meningitidis* ZnuD was produced in the membrane and solubilized with detergent prior to solving its structure (19). Thus, the nature of the soluble form of the ZnuD (with a polyhistidine tag and Mbp fusion partner) is uncertain and could potentially be a partially misfolded aggregate that is kept soluble by the presence of the N-terminal Mbps. This could be partially explain why the all loops (2, 5, 7, 11) hybrid antigen induced a more protective immune response than ZnuD (Figure 5), although we cannot exclude the increased focus on surface epitopes as an important factor.

The use of an N-terminal polyhistidine tag, Mbp protein with a TEV cleavage site proved to be useful for the isolation and preparation of the hybrid antigens, particularly those that formed inclusion bodies. Fortunately, neither this N-terminal region, nor the LCL scaffold induced an immune response that interfered with our ability to attribute the observed protection from infection to the displayed loops. This approach would be appropriate for further study of the impact of the various ZnuD loops on induction of a protective immune response. The previous study that introduced the hybrid antigen approach (21) selected several surface epitopes and two smaller surface loops that showed some utility in inducing an immune response that showed potential protection.

In this study, all of the individual surface loops were selected based on programs that predict B-cell epitopes and were able to induce partial protection from sepsis, as well as full protection with a combination of all four loops. However, it is currently uncertain whether the ability to induce an effective immune response would be limited to surface loops that were predicted to have the strongest B-cell epitopes or whether their selection could be achieved by other approaches. This system may be a logical one to further explore the parameters that can be used to optimize the induction of a protective immune response such as whether two or three loops will be sufficient in providing full protection and whether other loops from ZnuD can also provide protection.

Increases in IgG titers for most groups (Figure 4) were observed, however, the protection observed in the active immunization study (Figure 5) cannot be correlated to the titers observed on Day 35 for each group. This may be likely due to the design of the experiment as antibody titers were assessed against the immunizing antigen. While the protection observed can be attributed to the presence of the ZnuD loops on the scaffold, as the LCL scaffold on its own provided no protection, further experiments are needed to assess antibody titers to the native ZnuD to determine whether there is a correlate of protection between IgG titers and protection. Interesting, the active immunizations did not match the results of the passive immunization study. While the results show that antibodies are important for protection, the lack of total protection is likely due to the limited quantity of antibodies in the transfer. Further experiments may be needed to tease out the differences in results observed in the two models.

Although we were able to provide complete protection from infection in this study, it does not represent a viable option for development of a commercial vaccine product. The feasibility of preparing vaccine antigens for commercial application through refolding of inclusion bodies is questionable, thus alternate expression options need to be considered for production of hybrid antigens. In addition, an alternate scaffold from *A. baumannii* would be a more logical choice for developing a vaccine against this pathogen. Even with viable solutions for production of the hybrid antigens the utility of a vaccine against *A. baumannii* faces many challenges. It is not clear how one would select candidates for vaccination or whether it is feasible to mount a sufficient immune response to provide protection in the clinical setting. It would be difficult to initiate an immunization regimen early enough in identified populations prior to their increased risk of infection. Immunotherapy might be a more plausible option but would involve considerably more research for it to be practical. It would likely require the generation of hyperimmune sera or the production of several monoclonal antibodies against conserved protective epitopes which could be applied at high titer, preferably prophylactically for patients at risk of infection.

## Data Availability Statement

The raw data supporting the conclusions of this article will be made available by the authors, without undue reservation, to any qualified researcher.

## Ethics Statement

The animal study was reviewed and approved by Animal Care and Ethical Committee of Shahed University.

## Author Contributions

MQ was involved in the animal and immunological analysis experiments. SA was involved in the bioinformatics analysis. SC was involved in the design of the hybrids. AS and IR conceptualized the study. All authors were involved in the preparation of the manuscript.

### Conflict of Interest

AS is an inventor on a patent that describes the design and production of hybrid antigens and is a co-owner of Engineered Antigens Inc. The remaining authors declare that the research was conducted in the absence of any commercial or financial relationships that could be construed as a potential conflict of interest.
